# Evolutionary Dynamics of Pandemic Methicillin-Sensitive *Staphylococcus aureus* ST398 and Its International Spread via Routes of Human Migration

**DOI:** 10.1128/mBio.01375-16

**Published:** 2017-01-17

**Authors:** Anne-Catrin Uhlemann, Paul R. McAdam, Sean B. Sullivan, Justin R. Knox, Hossein Khiabanian, Raul Rabadan, Peter R. Davies, J. Ross Fitzgerald, Franklin D. Lowy

**Affiliations:** a Department of Medicine, Columbia University Medical Center, New York, New York, USA; b The Roslin Institute, University of Edinburgh, Edinburgh, Scotland, United Kingdom; c Department of Biochemistry and Molecular Biology, University of Melbourne, Parkville, Victoria, Australia; d Rutgers Cancer Institute of New Jersey, Rutgers University, New Brunswick, New Jersey, USA; e Department of Systems Biology, Columbia University, New York, New York, USA; f Department of Veterinary Population Medicine, University of Minnesota, St. Paul, Minnesota, USA; g Department of Pathology, Columbia University Medical Center, New York, New York, USA; University of Chicago Children’s Hospital; Skirball Institute of Biomolecular Medicine, New York University Medical Center

## Abstract

Methicillin-susceptible *Staphylococcus aureus* (MSSA) accounts for the majority of *S. aureus* infections globally, and yet surprisingly little is known about its clonal evolution. We applied comparative whole-genome sequencing (WGS) analyses to epidemiologically and geographically diverse ST398-MSSA, a pandemic lineage affecting both humans and livestock. Bayesian phylogenetic analysis predicted divergence of human-associated ST398-MSSA ~40 years ago. Isolates from Midwestern pigs and veterinarians differed substantially from those in New York City (NYC). Pig ST398 strains contained a large region of recombination representing imports from multiple sequence types (STs). Phylogeographic analyses supported the spread of ST398-MSSA along local cultural and migratory links between parts of the Caribbean, North America, and France, respectively. Applying pairwise single-nucleotide polymorphism (SNP) distances as a measure of genetic relatedness between isolates, we observed that ST398 not only clustered in households but also frequently extended across local social networks. Isolates collected from environmental surfaces reflected the full diversity of colonizing individuals, highlighting their potentially critical role as reservoirs for transmission and diversification. Strikingly, we observed high within-host SNP variability compared to our previous studies on the dominant methicillin-resistant *Staphylococcus aureus* (MRSA) clone USA300. Our data indicate that the dynamics of colonization, persistence, and transmission differ substantially between USA300-MRSA and ST398-MSSA. Taken together, our study reveals local and international routes of transmission for a major MSSA clone, indicating key impacts of recombination and mutation on genetic diversification and highlighting important ecological differences from epidemic USA300. Our study demonstrates extensive local and international routes of transmission for a major MSSA clone despite the lack of substantial antibiotic resistance.

## IMPORTANCE

Unlike methicillin-resistant *Staphylococcus aureus* (MRSA), surprisingly little is known about the clonal evolution of methicillin-susceptible *S. aureus* (MSSA), although these strains account for the majority of *S. aureus* infections. To better understand how MSSA spreads and becomes established in communities, we applied comparative bacterial whole-genome sequencing to pandemic ST398-MSSA, a clone of clinical importance affecting humans and livestock in different geographic regions. Phylogeographic analyses identified that ST398-MSSA spread along local cultural and migratory links between parts of the Caribbean, North America, and France, respectively. We observed high within-host SNP variability compared to our previous studies on the dominant MRSA clone USA300. Our data indicate that the dynamics of colonization, persistence, and transmission differ substantially between USA300 MRSA and ST398 MSSA.

## INTRODUCTION

*Staphylococcus aureus* causes the majority of skin and soft tissue infections (SSTIs) and is also frequently associated with invasive disease, such as bloodstream infections or pneumonia (1). Over the past few decades, novel methicillin-resistant *S. aureus* (MRSA) genotypes have emerged due to the acquisition of variants of the staphylococcal cassette chromosome *mec* (SCC*mec*) element. Only a minority of emergent MRSA clones have disseminated globally and have caused epidemic waves of health care-associated (HA), community-associated (CA), or, more recently, livestock-associated (LA) *S. aureus* infections.

The advent of high-resolution whole-genome sequencing (WGS) has allowed the reconstruction of the evolution of several major MRSA clones such as multilocus sequence types (STs) ST239, ST22, ST30, ST80, and ST8/USA300 by sequencing of large data sets (2–7). Collectively, these studies have documented how specific antibiotic selective pressures, as well as interactions with human hosts, have shaped the recent evolutionary history of these MRSA clones. While these studies have included a selected number of methicillin-susceptible *S. aureus* (MSSA) isolates (7), the emergence, evolution, and transmission of major MSSA lineages have been largely disregarded. MSSA, however, remains of considerable public health importance as it accounts for the majority of health care- and community-based *S. aureus* infections throughout the world (8). In a possible reversal of past trends, it has been suggested that MSSA now accounts for higher numbers of HA-*S. aureus* (HA-SA) infections than does MRSA (9). Although the clonal backgrounds of MSSAs are highly diverse, a number of dominant pandemic MSSA clones have been identified (8, 10, 11). Major MSSA lineages overlap the dominant circulating MRSA clones and include ST30, ST5, ST8, and, more recently, ST398 (12). ST398 was first described as both MSSA and MRSA among pig farmers in France (13, 14). Since the original description, MRSA ST398 has quickly spread among pigs and other types of livestock and has been associated with MRSA infections in farmers (15, 16). MRSA ST398 strains are thought to spread only infrequently beyond the immediate animal and farm contacts (17–19).

In parallel, animal-independent human colonization and infections with ST398 MSSA have emerged and have now been encountered worldwide, including in several European countries (14, 20, 21), the Caribbean (22), and the northeastern United States (23, 24). The prevalence of ST398 MSSA appears to be particularly high in parts of China, where this clone accounts for almost 20% of skin and soft tissue infections (SSTIs) (25). This lineage has been notably absent from other regions such as large parts of the United States (11), although it has sporadically been reported in pigs and veterinarians in the Midwest (26). ST398 MSSA strains are encountered as both CA and HA pathogens (27, 28). Remarkably, a number of studies from such diverse settings as community households, a jail holding tank, and an intensive care unit have suggested that ST398 MSSA might be uniquely transmissible between humans (24, 27, 29). In addition to the distinct epidemiology, important genomic differences between human MSSA and LA-MRSA ST398 include a large repertoire of mobile genetic elements (MGEs) (30) and tetracycline resistance and lack of a unique SA3*int* β-hemolysin converting phage (21, 24, 31). In contrast, most of the human-associated ST398 isolates harbor resistance to macrolides and are *spa* type t571. WGS analyses indicated a possible human origin for LA-ST398, followed by the emergence of methicillin resistance driven by antibiotic pressure in animal feeds (31). A more recent quantitative time-scaled phylogeny, however, indicated that both human MSSA and LA-CC398 emerged in parallel around 1970 (32).

To further delineate the basis of the wide geographic distribution of ST398 MSSA, we investigated the macro- and microevolution of this clone by comparative whole-genome sequencing of epidemiologically linked isolates from social networks in New York City, farms in the midwestern United States, and geographically distinct Caribbean islands. Our analysis provides insights into the recent adaptation and international spread of this highly successful MSSA clone.

## RESULTS

### Phylogenetic reconstruction of human ST398 MSSA isolates.

**FIG 1:**
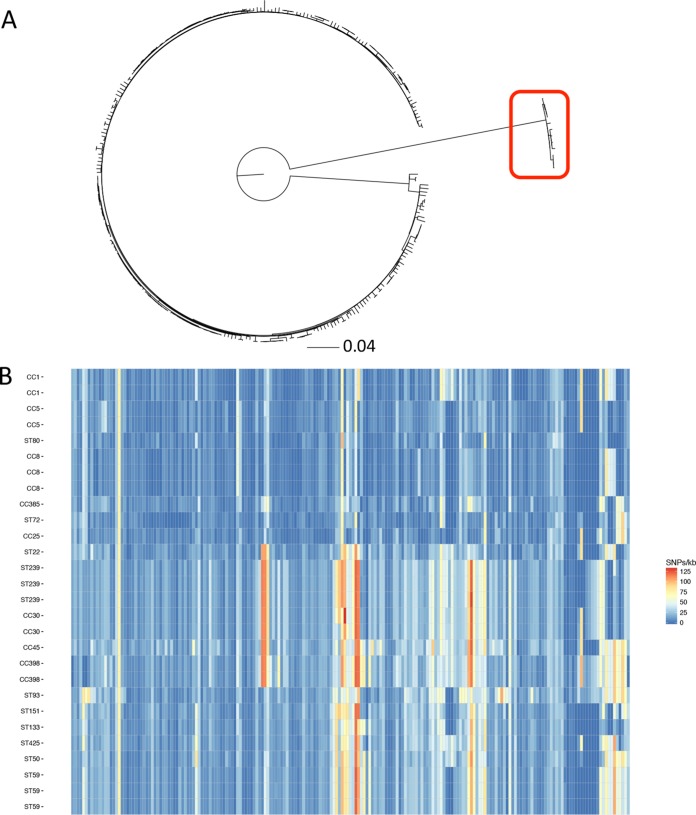
Phylogeny of ST398 reveals large-region recombination. (A) Maximum likelihood phylogenetic tree of ST398 isolates, rooted by midpoint, indicates that a subset of isolates (12 of the 14 isolates from veterinarians and pigs) is distinct. (B) Heat map of alignment of the ~250-kb region of recombination at *ori* indicates a mosaic origin from multiple clonal complexes (CC385, CC25, CC1, CC5, CC97, and CC8) rather than from a single clone. Single-nucleotide polymorphism density across the large recombinant region spanning origin of replication in the veterinary/animal strains with recombinant region (reference genome coordinates start at 2557000 and end at 58000). Each cell represents a block of 1,000 bp, and shading indicates density of SNPs in that region, with red indicating greater divergence. A representative selection of samples from both animals and humans was included.

In order to determine the evolutionary history of human ST398 MSSA, we determined full-genome sequences of 288 *spa*-clonal complex (CC) t571/ST398 isolates (Fig. 1; see also Table S1 in the supplemental material). These isolates were selected from community-based studies on the transmission of *S. aureus* in Northern Manhattan (NM) and the Bronx (22, 24, 28, 33) and from t571-MSSA-colonized veterinarians and pigs in Minnesota. To increase the genetic, temporal, and geographic diversity in the sample set for comparative sequence analyses, we also included previously published ST398 MSSA and MRSA sequences from international HA- and LA-CC398 collections (31).

10.1128/mBio.01375-16.5 TABLE S1  Summary of isolates and accession numbers. Download TABLE S1, XLSX file, 0.05 MB. Copyright © 2017 Uhlemann et al. 2017 Uhlemann et al. This content is distributed under the terms of the Creative Commons Attribution 4.0 International license.

Mapping to the ST398 reference genome and subsequent phylogenetic reconstruction revealed a distinct long branch length for 12 of the 14 isolates from veterinarians and pigs. These samples harbored a unique ~250-kb region at the *ori* compared to the other isolates (Fig. 1A). Comparison of this region and previously published reference genomes revealed that this sequence is of mosaic origin, suggestive of multiple large-scale recombination events (34) (Fig. 1B). The remainder of the sequence in these 12 isolates was consistent with an ST398 clonal background.

**FIG 2:**
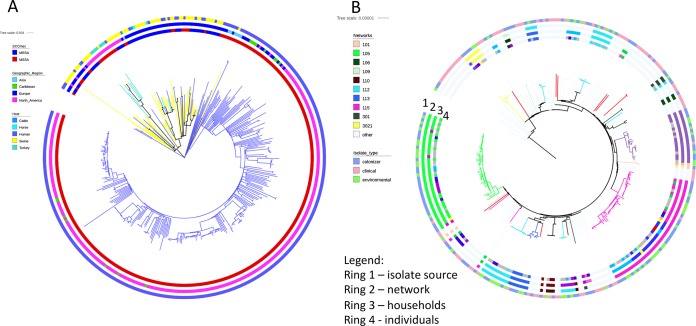
ST398 phylogeny and emergence of ST398. (A) Maximum likelihood phylogenetic tree of ST398 indicates that isolates from Northern Manhattan form a distinct clade of human MSSA isolates. The outer ring and branches are colored by host origin, the middle ring indicates geographic region, and the inner ring shows the presence or absence of the SCC*mec* element. (B) ML phylogenetic tree indicates mapping of isolate source (outer ring 1) and social networks (ring 2 and colored branches). Ring 3 shows households within networks (index household color matches network). Inner ring 4 highlights the overlap of isolates collected from different individuals and environmental surfaces within network households (index color matches network color). Gray indicates environmental isolates. Light blue in circles 2 to 4 represents nonnetwork isolates.

After exclusion of these 12 isolates, we removed mobile genetic elements (MGEs), predicted recombinant regions from the alignment, and identified 9,843 single nucleotide polymorphisms (SNPs) in comparison with the ST398 MSSA reference sequence (24). These “core” SNPs were used to construct a maximum-likelihood phylogenetic tree (Fig. 2A).

Mapping the geographic and host origin of isolates onto the phylogenetic tree showed clades with significant associations with both host and location (*P* < 0.01 for both traits, Slatkin-Maddison test; see Fig. S1 in the supplemental material). Isolates from the NM collection were relatively closely related and clustered separately from previously published LA-MRSA and most human CC398 strains from Europe (31) (Fig. 2A). The two isolates without evidence of large-scale recombination, collected from veterinarians in Minnesota, clustered with LA-CC398 isolates rather than with NM ST398 isolates.

10.1128/mBio.01375-16.1 FIG S1  Results of Slatkin-Maddison tests for trait association. (A) Expected distribution of host transitions across phylogeny with 10,000 permutations. Red line indicates observed number of transitions. (B) Expected distribution of location transitions across phylogeny with 10,000 permutations. Red line indicates observed number of transitions. Download FIG S1, TIF file, 1.5 MB. Copyright © 2017 Uhlemann et al. 2017 Uhlemann et al. This content is distributed under the terms of the Creative Commons Attribution 4.0 International license.

Dominican isolates were interspersed with the majority of the isolates from the largely Dominican NM neighborhood. ST398 isolates from Martinique formed a distinct clade that also contained previously reported sequence from France (31) and several NM isolates (Fig. 2A). Within the NM clades, ST398 isolates from infections were interspersed with colonizing isolates (Fig. 2B). Importantly, none of the previous ST398 isolates from hospital-associated infections (28), which had been thought to represent a potential nosocomial ST398 outbreak, were closely related.

### Time scale of the emergence of human CC398 in Northern Manhattan and phylogeographic reconstruction.

**FIG 3:**
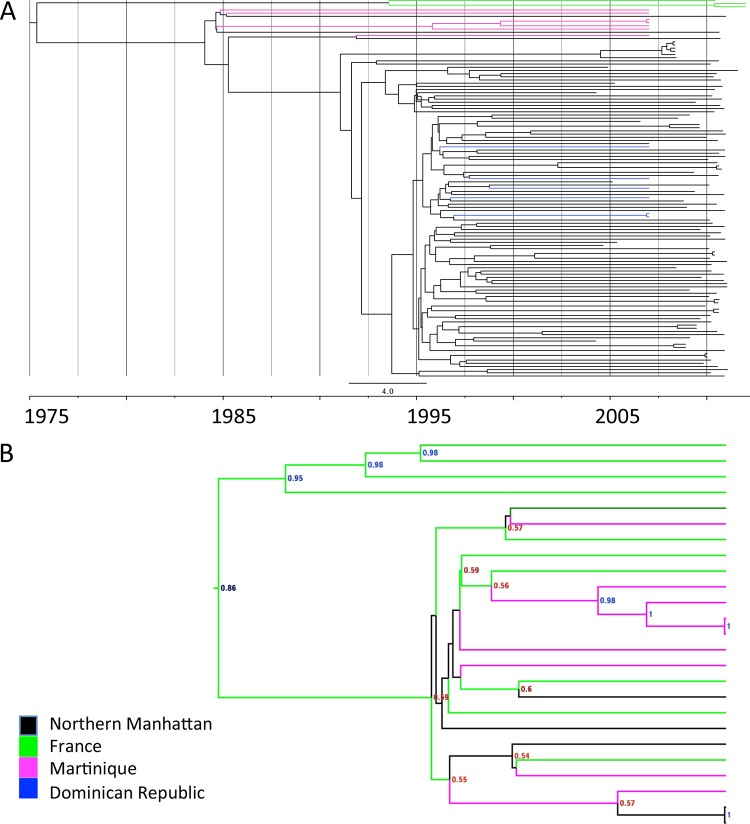
Bayesian phylogenetic reconstruction of ST398 isolates. (A) Maximum clade consensus tree, estimated from core genome SNPs of 119 isolates. Branches are scaled with time (months/years). Colors of internal and terminal branches indicate region of origin. (B) Phylogeographic spread of ST398 isolates between France and Martinique.

To further reconstruct the evolution and geographic spread of the human-associated ST398 lineage, we selected isolates that showed evidence for clocklike evolution (*n* = 119) for Bayesian phylogeny. These included all geographically diverse isolates from Martinique and the Dominican Republic (DR) (*n* = 14) and Minnesota (*n* = 2) and from NM infectious isolates (*n* = 69) and colonizing isolates (*n* = 11) and a limited number of network isolates (*n* = 23). We estimated a substitution rate at 1.49 × 10^−6^ substitutions per site per year in the BEAST analysis (95% highest posterior density [HPD] interval = 9.44 × 10^−7^ to 1.97 × 10^−6^). This rate is largely consistent with rates that have been previously reported for other *S. aureus* clones and for the CC398 lineage (5, 32) and is consistent with the estimate obtained from a root-to-tip regression of the full data set (1.47 × 10^−6^ substitutions per site per year) (see Fig. S2 in the supplemental material). The median time of clonal emergence was estimated around 36.6 years ago (95% HPD, 24.3 to 54.6) with a most recent common ancestor (MRCA) of the ST398 MSSA lineage in 1976 (Fig. 3). These findings are in accordance with a prior study including CC398 LA and HA clades that estimated their divergence around 1971 (32).

10.1128/mBio.01375-16.2 FIG S2  Root-to-tip regression analysis. Download FIG S2, PDF file, 0.01 MB. Copyright © 2017 Uhlemann et al. 2017 Uhlemann et al. This content is distributed under the terms of the Creative Commons Attribution 4.0 International license.

This scaled phylogeny also indicates that ST398 isolates from Martinique diverged around 1988 (95% HPD, 1972 to 1992). In contrast, Dominican isolates were embedded within several clades of NM isolates (Fig. 3A). To further investigate the geographic spread of CC398 between the French overseas department Martinique and France, we carried out a phylogeographic reconstruction including French isolates from the Price et al. study (31) (Fig. 3B). This analysis revealed that the closest relatives of the Martinique strains are mostly from France, although not all nodes have high confidence in the predicted origin, which may reflect an undersampling of the strains of interest.

### ST398 variable genome.

MGEs are important contributors to virulence in *S. aureus* and to distinguishing livestock- and human-associated clades (24, 30, 31, 35). While LA-CC398 harbors an extensive repertoire of *S. aureus* phages, only a limited number of phages have been observed in human-CC398. Consistent with prior studies, mapping of prophages revealed that prophage SA3*int* was present in all human ST398 isolates, whereas prophages SA4*int* and SA8*int* were universally absent (see Fig. S3 in the supplemental material). Although only infrequently present in this sample collection, the Panton-Valentine leukocidin (PVL)-carrying phage SA2*int* was present in 12.5% (9/70) of infectious isolates but only 1.5% (2/139, *P* = 0.0016) of colonizing isolates (Fig. S3). In one household persistently colonized with two distinct ST398 clades, of which initially only one was carrying SA1*int*, we subsequently detected this phage in both clades, suggesting exchange of SA1*int* between isolates. Isolates from veterinarians and pigs in Minnesota, including those with the large-scale recombination, lacked SA3*int* and predominantly harbored SA6*int* (*n* = 8), consistent with an LA-ST398 origin.

10.1128/mBio.01375-16.3 FIG S3  Accessory genome diversity and stop codon in YeeQ in ST398. Colored branches indicate the presence (pink) or absence (green) of a stop codon in YeeQ. Circles (starting with inner circle): (1) human versus livestock origin, (2) Sa2*int*, (3), Sa3*int*, and (4) presence of either Sa1*int*, Sa5*int*, Sa6*int*, or Sa7*int*. Download FIG S3, TIF file, 1.5 MB. Copyright © 2017 Uhlemann et al. 2017 Uhlemann et al. This content is distributed under the terms of the Creative Commons Attribution 4.0 International license.

**FIG 4:**
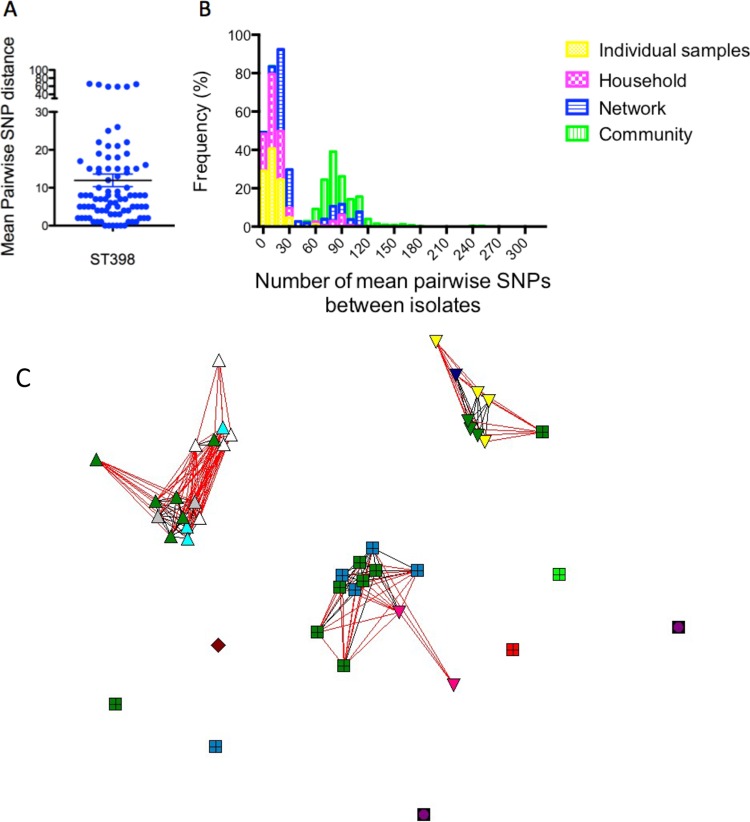
(A) Pairwise SNP comparisons between multiple samples taken from different body sites in individuals. (B) Mean pairwise SNPs between isolates from individuals (yellow), households (pink), or networks (blue) or between isolates from different community households (green). (C) SNP distance-based mapping of ST398 isolates from the largest social network indicates microevolution of separate clusters and frequent exchange between individuals and the environment and between households. Each symbol represents a single isolate, and isolates are clustered by their pairwise SNP distance. Each of the four embedded households is represented by a different shape; each individual is shown in a different color. All dark green symbols represent environmental samples. Isolates connected by red lines are separated by up to 10 pairwise SNPs. Nonconnected isolates are less than 25 SNPs different and are proportional to the number of SNPs.

We did not detect SNPs that uniquely defined the NM ST398 clade. However, a stop codon in *yeeR*, coding for a putative membrane protein, was uniquely associated with a major clade of the CC398 LA-MRSA isolates (Fig. S3).[Supplementary-material figS4]

10.1128/mBio.01375-16.4 FIG S4  ST398 populations within households and networks are under selective pressure. Frequency distribution of Tajima’s *D* across households (circle) and networks (square). Download FIG S4, PDF file, 0.03 MB. Copyright © 2017 Uhlemann et al. 2017 Uhlemann et al. This content is distributed under the terms of the Creative Commons Attribution 4.0 International license.

### Microevolution of ST398 within community households and networks.

To investigate the transmission properties of ST398 in the community, we carried out a network study of individuals previously identified as ST398 positive. We enrolled 15 networks that encompassed 348 participants from 81 different households. Thirty-three individuals from 20/81 (25%) households were colonized with t571-ST398 MSSA. ST398-colonized and noncolonized individuals were comparable in their demographics and comorbidities (see Table S2 in the supplemental material). They differed by only an increased household size and a decreased household density in the ST398 group as well as a higher frequency of recent SSTIs (16% versus 7%, *P* = 0.04). Fourteen participants (42%) remained longitudinally colonized with ST398 MSSA for the duration of their study participation of up to 12 months.

10.1128/mBio.01375-16.6 TABLE S2  Demographic characteristics of ST398 network participants. Download TABLE S2, XLSX file, 0.03 MB. Copyright © 2017 Uhlemann et al. 2017 Uhlemann et al. This content is distributed under the terms of the Creative Commons Attribution 4.0 International license.

The majority of ST398 isolates collected from the same individual, household, or network clustered closely together in phylogenetic analyses as illustrated by short terminal branches (Fig. 2B). However, there were several exceptions in which select individuals (*n* = 2), households (*n* = 6), or networks (*n* = 5) harbored at least two highly distinct ST398 clades. Notably, in one large network with four ST398-colonized households, each harbored distinct ST398 subclades.

To further define the clonal diversity of individuals, households, and networks, we estimated the pairwise SNP distances between isolates. We found that the median pairwise SNP distance of isolates on the same individual was 8 (range, 0 to 80), that within households was 13 (range, 0 to 105), that within networks was 25 (range, 3 to 114), and that in the community was 82 (range, 0 to 312) (Fig. 4).

We then applied these SNP diversity values to define and investigate transmission. While isolates mainly clustered by individual and household, there was extensive overlap of genotypes between individuals and households and across four networks, suggesting frequent exchange. Environmental isolates reflected most of the diversity present on individuals. Overall, these data indicate that closely interacting individuals share a cloud of isolate diversity that extends across households and networks (Fig. 2B and 4C).

These SNP-based diversity measures were significantly higher than those in our previous study on USA300-MRSA transmission in community households (individuals, 1.4 [range, 0 to 9], and households, 4 [range, 0 to 23]) (5), although substitution rates were comparable for the two clones (5, 32). We explored several possibilities for the relatively high SNP diversity. First, we compared longitudinal isolates that were collected 6 months apart (*n* = 38). These follow-up samples did not have excess mutations based on the estimated substitution rate of 4 SNPs per year. We also did not observe mutations or premature stop codons in candidate genes of hypermutability such as *mutL*/*mutS*.

To explore deviations from neutral evolution within households and networks, we performed Tajima’s *D* neutrality test (36). Following the method of Tajima (36), we assumed beta-distributed confidence limits for *D*. All but one household and network yielded negative *D* values (see Fig. S2). We identified significant nonrandom evolutionary processes in two networks: network 105 (*D* = −1.95, *P* < 0.05) and network 114 (*D* = −2.25, *P* < 0.01). Only one household in network 114 had a sufficient number of samples to recapitulate the network’s significant results (*D* = −2.01, *P* < 0.05). Although we did not find values of *D* to be significant across genes, we found one pathway in network 105 and eight pathways in network 114 corroborating significant values of *D* from whole-genome sequences (see Table S3 in the supplemental material). We, however, found no evidence for purifying or positive selection, as the ratios of nonsynonymous to synonymous substitutions (*dN*/*dS*) were not significantly different from 1 across the study and within network-specific lineages. This suggests that negative *D* values are possibly due to the presence of rare alleles at low frequencies that accumulated during a recent selective sweep or an expansion after a bottleneck.

10.1128/mBio.01375-16.7 TABLE S3  Tajima *D* calculations for household and networks. Download TABLE S3, XLSX file, 0.1 MB. Copyright © 2017 Uhlemann et al. 2017 Uhlemann et al. This content is distributed under the terms of the Creative Commons Attribution 4.0 International license.

## DISCUSSION

There is a lack of information on the evolution and spread of dominant MSSA lineages. This is one of the first longitudinal studies to investigate the evolution of the uniquely host-species-adaptable ST398 MSSA clone in the community using a combined social network and genomic approach. Similarly to recent household studies on USA300 MRSA (5, 37), we found that households served as major sites for ST398 MSSA transmission. However, by taking advantage of our network enrollment strategy, we were able to demonstrate frequent spread of these isolates within social networks as evident by overlapping SNP distances. We also found evidence for long- and short-distance geographic migration of ST398. First, isolates from infections that occurred during hospitalizations did not differ from colonizing isolates and were embedded within the clade of NM community isolates. This observation highlights the potential role of common community commensals in hospital-associated *S. aureus* infections. Second, isolates from France and the French overseas department Martinique were more closely related to evidence for spread from France to Martinique. French tourists account for the majority of visitors (75%) each year to Martinique (38). Bayesian phylogeographic reconstruction supported the root of Dominican ST398 isolates in Northern Manhattan and suggested subsequent spread to the Caribbean island, which is a frequent travel destination from this community (22). These links may help explain the disparate distribution of this clone in different geographic regions.

We observed a relatively high and overlapping SNP diversity within individuals, environmental surfaces, households, and networks. In our previous study on USA300 MRSA transmission in the same community, the SNP diversity was much lower on individuals and in households. However, the substitution rates observed here for ST398 as well as previously published rates for both clones (5, 32) are comparable. When analyzing longitudinal isolates collected from the same individual, we did not find any evidence for an increased accumulation of SNPs in ST398 isolates over this time period. We also did not detect SNPs in genes associated with hypermutability such as *mutL* or *mutS*.

Alternatively, differences in SNP diversity between lineages might be a direct reflection of long-term persistence of ST398-MSSA over years versus short-term colonization with USA300 in community households. This might be attributable to the increased virulence potential of USA300, the predominant CA-MRSA strain and major cause of SSTIs in the United States (39). Alam et al. (37), however, suggested long-term persistence of USA300 in households in Chicago and Los Angeles ranging between 2.3 and 8.4 years. In our study, we did not detect a temporal signal within households due to the relatively large number of SNPs at each sampling time point, thus precluding timing of persistence. While both studies provide evidence for long-term persistence of successful *S. aureus* clones, it should be noted that these stand in contrast to rapid changes in individual colonization based on *spa* typing alone (40). The observed high SNP diversity in individuals has direct implications for epidemiological transmission studies, including in the health care setting, when patients become infected with their colonizing bacteria. Differences in SNP diversity between *S. aureus* clones such as ST398 and USA300 or other bacterial clones may need to be considered in defining linkages during hospital outbreaks.

Although we found no evidence for presence of selection from the *dN*/*dS* estimates, Tajima’s *D* neutrality test suggests deviations from neutral evolution in households and networks. Negative Tajima *D* values indicated that the number of observed genetic differences (theta) is smaller than the expected value (pi), consistent with the presence of rare alleles at low frequencies, possibly due to a recent selective sweep or expansion after a bottleneck such as after transmission and adaptation to new colonization sites. Our study provides preliminary evidence that this might be driven by accumulation of SNPs in specific functional pathways. Despite differences in measuring genetic diversity between our study and that by Alam et al., both investigations observed that the number of observed genetic differences (theta) is smaller than the expected (pi), resulting in negative Tajima *D* values.

In a prior study on differences between human MSSA and LA-CC398 MRSA, we observed pseudogenes and variations in genes encoding surface adhesins in LA isolates but not in human samples (24). This further translated into impaired binding of LA-ST398 to human keratinocytes *in vitro*, whereas both human and LA isolates were able to adhere to porcine keratinocytes. Many of these mutations or insertions and deletions were detected in repetitive elements of these genes. Short-read sequences obtained by Illumina sequencing here preclude a thorough and reliable assessment of these regions.

We detected evidence for a large-scale recombination within a subset of ST398 MSSA isolates obtained from pigs and swine veterinarians in three Midwestern states. This is at least the second report of recombination in this clonal lineage (31). However, in contrast to large-scale recombinations previously described in ST398 and other *S. aureus* clonal complexes, the origin of this ~250-kb region cannot be attributed to a single clonal lineage. This indicates either recombination from a not-yet-identified novel sequence type or indeed mosaic acquisition of several regions from a diversity of clonal complexes (CCs). Homologous recombination might provide additional means of adapting to a novel host environment (34, 41).

Several limitations to our study need to be considered. Despite efforts to enroll complete social networks, we were able to recruit only about one-third of named close social contacts, introducing possible selection bias. Follow-up data were limited as individuals had moved or were not willing to participate any longer. Cost precluded a complete assessment of environmental samples. Phylogeographic reconstruction is vulnerable to sampling bias. More comprehensive sampling from other geographic origins may have changed the inferred origin of ST398.

In conclusion, we reconstructed the micro- and macroevolution of ST398-MSSA, a clone that is uniquely successful in diverse regions despite the lack of an array of antimicrobial resistance genes. Phylogeographic reconstruction suggests recent spread from France to Martinique and from NM to the DR, in accordance with cultural links and travel destinations (22), rather than spread within the Caribbean. We provide evidence for an extensive cloud of diversity of ST398-MSSA shared between individuals and the environment in households, extending to social networks, which was distinct from USA300. Recognizing these differences between clonal lineages has important implications for outbreak investigations, including in the clinical setting.

## MATERIALS AND METHODS

### Ethics statement.

This study was reviewed and approved by the Institutional Review Board of Columbia University Medical Center. Written informed consent was obtained from each individual before participation. Parental consent was required for the participation of children <18 years old. Pediatric assent was obtained from those capable of providing it.

### Study population, demographics, and sample selection.

As part of a community study on *S. aureus* transmission in Northern Manhattan, we implemented a cluster network analysis of ST398 transmission between September 2010 and March 2013. Individuals who had previously tested positive for ST398 colonization or infection were eligible to enroll (28, 42). These index participants were asked to complete a structured questionnaire and provide information on their daily contacts and the nature and frequency of these interactions. In addition, swabs were collected from the anterior nares, throat, and groin from consenting participants (Becton, Dickinson Culturette Systems). A standardized set of environmental items was also sampled with sterile premoistened swabs in all households: doorknobs, television remote control, living room light switch, toy, couch or bed, computer or radio, house phone or index cellular phone, bathroom sink, kitchen, and appliance handle (33).

A snowball enrollment strategy was used to identify contacts of index participants. Named household and outside-household contacts of the index were approached and after providing informed consent underwent the same study procedures. Contacts were further defined by their type of relationship (e.g., family, work, school, sports, or other social life contacts). Recruitment occurred in waves, such that contacts of study participants were asked to participate in the study, as were the contacts of these newly recruited contacts. Follow-up household visits were carried out twice: between 3 and 6 or 6 to 12 months after enrollment, respectively. We enrolled individuals from 15 networks that consisted of 958 named members. Of these, 348 were enrolled and 273 individuals agreed to complete the questionnaire. We compared demographic variables and risk factors for *S. aureus* infections between individuals colonized with ST398 and those colonized with other *S. aureus* isolates or not colonized. Data were analyzed using SAS 9.1 software (SAS Institute, Cary, NC).

Culture swabs were incubated overnight at 37°C in high-salt 6.5% broth and plated onto mannitol salt agar (Becton, Dickinson) for 48 h at 35°C. Positive mannitol-fermenting yellow colonies were isolated onto 5% sheep blood agar plates (Becton, Dickinson), and single colonies were selected for further analysis. *S. aureus* was identified by a coagulase and protein A detection kit (Murex Staphaurex). *S. aureus*-positive isolates were genotyped by *spa* sequencing using Ridom-staph software (24, 25). Of 2,590 swabs, we identified 690 *S. aureus* isolates. Based on *spa* typing and Spa clustering, 175 isolates belonged to Spa-CCt571, consistent with CC398.

Of these, we selected 153 isolates, including all human-colonizing isolates (*n* = 96) and a subset of environmental isolates (57/79) for sequencing. We also selected an additional 135 ST398 isolates from previous studies on *S. aureus* infection and colonization in Northern Manhattan and the Bronx as follows: (i) MSSA infections between 2007 and 2011 (*n* = 69) (24, 28), (ii) infectious isolates from the Dominican Republic and Martinique (*n* = 14) (22), (iii) community studies on colonization between 2003 and 2010 (*n* = 38) (23, 33, 42), and (iv) colonizing isolates from pigs in Minnesota and swine veterinarians in Minnesota, Illinois, and Indiana (*n* = 14) (26). In total, we obtained 288 novel sequences. Previously published sequences from international studies were also included in phylogenetic analyses (31, 32).

### WGS and detection of SNPs in the core genome.

*S. aureus* genomic DNA was extracted using the QIAamp DNA minikit (Qiagen), and unique index-tagged libraries were generated. WGS was carried out using the Illumina HiSeq2000 with 100-base paired-end reads. Paired-end reads were mapped against the core chromosome of the CC398 reference genome (GenBank accession number CP003045.1 [24]) using the Burrows-Wheeler Aligner (BWA) with the Smith-Waterman algorithm disabled as described previously (4). The same approach was used to assemble publicly available CC398 short reads. A core genome alignment was created from the consensus sequences, with the core genome defined as nucleotide sites shared by all sequences (alignment columns that did not contain a gap character or unknown nucleotide identity). Unmapped reads and sequences that were not present in all genomes and MGEs were not considered part of the core genome, and therefore, SNPs from these regions were not included in the phylogenetic analysis. For the ~250-kb region at the *ori*, we mapped sequence reads against known animal and human *S. aureus* reference genomes. Further regions that may be affected by recombination were tested using BratNextGen and removed. This ensured a curated high-quality data set for subsequent phylogenetic analysis comprised of 1,826,995 bp.

### Phylogenetic analyses.

Maximum-likelihood phylogenetic reconstruction of the CC398 isolate sequences was performed using RAxML v7.4.0 with the general time-reversible (GTR) model of nucleotide substitution and 1,000 bootstrap replicates (43). The resulting phylogenetic trees were rooted at the midpoint between the most divergent taxa in the tree. These analyses included either (i) the entire data set including previously published MSSA and MRSA genomes (376 isolates, 9,843 SNPs) or (ii) human ST398 MSSA sequenced in this study. Association of traits (host and location) with clades within the phylogeny was tested using the method described by Slatkin and Maddison (44). Trees were annotated using iTOL (45).

### Time-scaled phylogenetic analysis.

To investigate the temporal signal present in the maximum-likelihood phylogeny, the tree and dated tips were loaded into Path-O-Gen (http://tree.bio.ed.ac.uk/software/pathogen/) to identify isolates where the assumption of a molecular clock is not valid. A subset of 119 clinical isolates from the Manhattan clone displayed evidence of clocklike evolution and were used to infer the substitution rate, time of emergence, divergence dates, and phylogeographic spread, applying Bayesian methods implemented in the BEAST v1.7.5 package (46). BEAST was run for 100 million generations sampling every 10,000 states using the HKY substitution model; defined tip dates as in day, month, and year of isolation; and a strict, exponential relaxed, and lognormal relaxed molecular clock with constant size coalescent or Bayesian skyline coalescent, respectively. Each model was run three times, and good convergence of chains and effective sample size (ESS) values were inspected using Tracer v1.5. A marginal likelihood estimation (MLE) using path sampling and steppingstone sampling for each run was carried out to compare the different combinations of clock and tree models (47, 48). The MLE was then used to assess the best-fitting model for the data set by calculating the Bayes factor. For the used data set, the strict skyline model provided the best fit. LogCombiner v1.7.5 was used to remove states before the burn-in and then to combine the trees from the multiple runs. A maximum clade credibility (MCC) tree from the combined trees was obtained using TreeAnnotator v1.7.5. To investigate transmission dynamics of *S. aureus* isolates from Martinique, discrete trait analysis was performed on the Martinique-associated clade, to determine the probability of the ancestral location at each node. BEAST was run for 100 million generations, sampling every 10,000 states, using the HKY substitution model, with the clock rate fixed at 1.

### *In silico* detection of MGEs.

Sequence reads were assembled *de novo* into contigs using Velvet v 1.0.12 (49) and Velvet Optimizer and were used to determine the presence and absence of MGEs. Prophages were classified according to their defined integrase type (50).

### Transmission and molecular evolution analyses.

We used pairwise single-nucleotide polymorphism (SNP) distances as a measure of genetic relatedness between isolates. Network relationships were visualized using Ucinet (51). We calculated Tajima’s *D* test to compare the population mutation rate to the pairwise nucleotide distance within households and networks to determine whether the observed frequency of segregating mutations matched the expected frequency under the standard neutral model. We implemented calculation of Tajima’s *D* in Matlab and assessed confidence intervals for *D* assuming the beta distribution as formalized by Tajima in 1989 (36). We calculated Tajima’s *D* for households and networks for which at least 4 samples were available. We also calculated Tajima’s *D* for genes and pathways based on the annotation of each genomic position using PATRIC’s database for *Staphylococcus aureus* subsp. *aureus* 71193 (GenBank accession no. CP003045.1). We obtained estimates for ω, the *dN*/*dS* ratio, for the community study isolates as well as each network, based on a branch-site model (52) as implemented in the PAML package (53). For this analysis, we concatenated 778 protein-coding genes, which were found mutated in at least two isolates. To assess the presence of selection, we computed the likelihood-ratio test statistics for the estimated ω values versus fixed ω = 1, using a chi-square test (54).

### Accession number(s).

Sequence data are deposited in the European Nucleotide Archive (study accession number PRJEB12818).
