# Supplementary figures and images for: Evolutionary Dynamics of Pandemic Methicillin-Sensitive Staphylococcus aureus ST398 and Its International Spread via Routes of Human Migration
Source: mBio. 2017 Jan 17;8(1):e01375-16. doi: 10.1128/mBio.01375-16 (PMC5241395; doi:10.1128/mBio.01375-16)

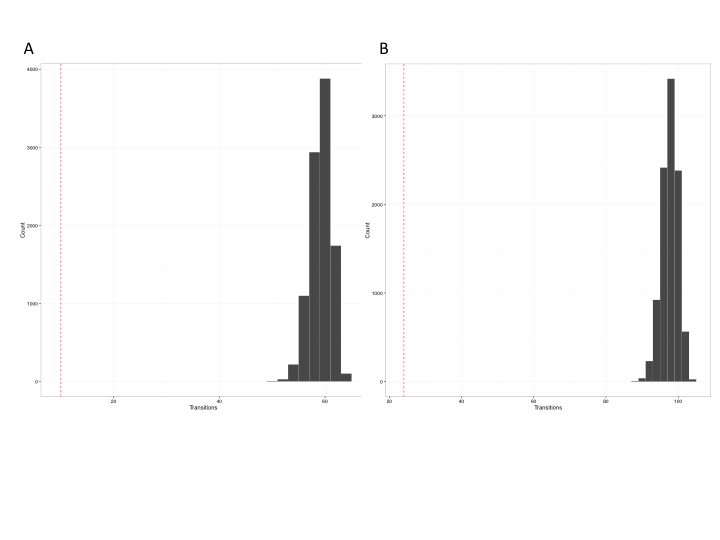

Supplement: FIG S1 [file mbo001173136sf1.tif]

Scatter plot of isolation date and root to tip divergence  
with regression line for NM398

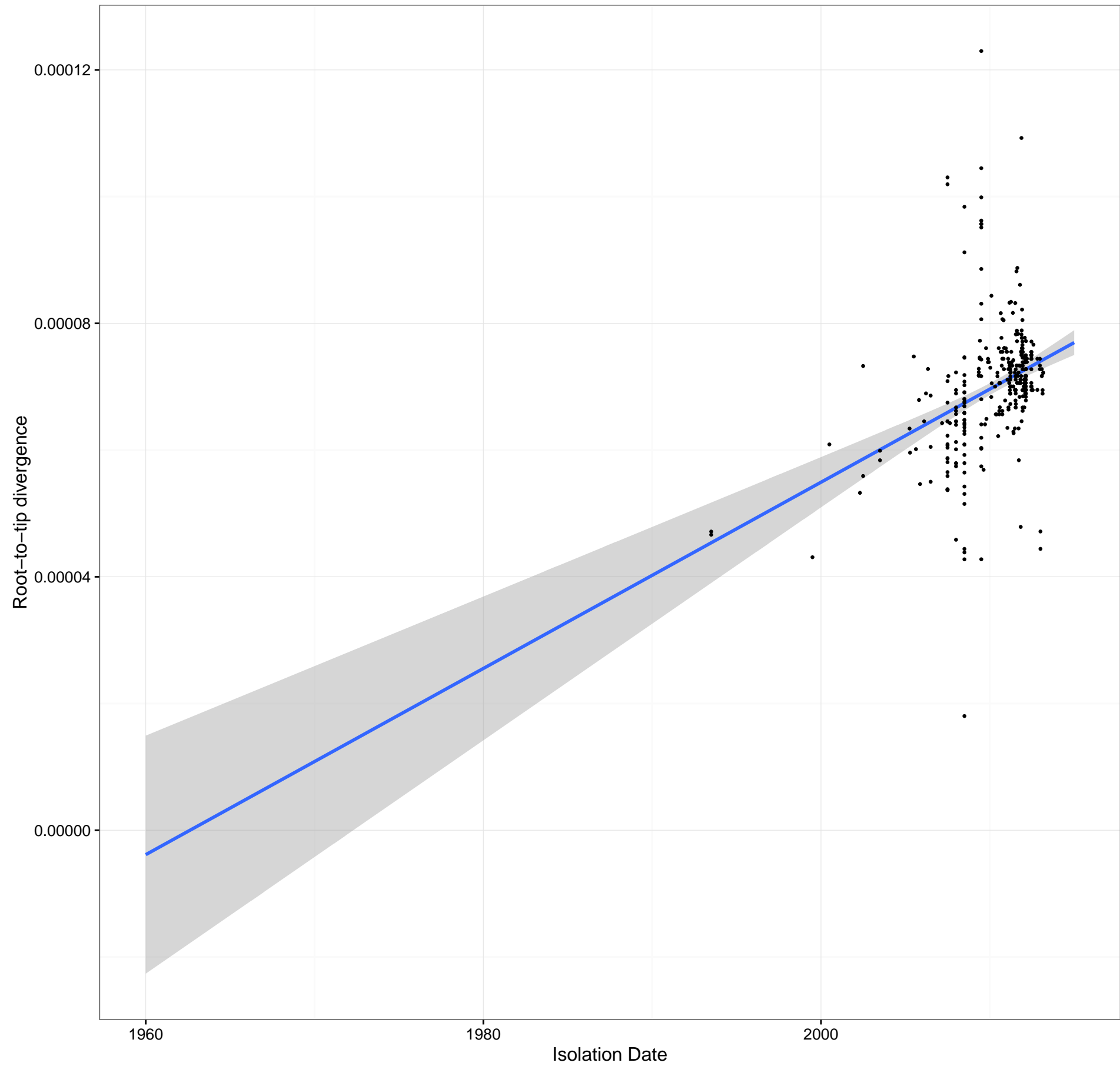

Supplement: FIG S2 [file mbo001173136sf2.pdf]

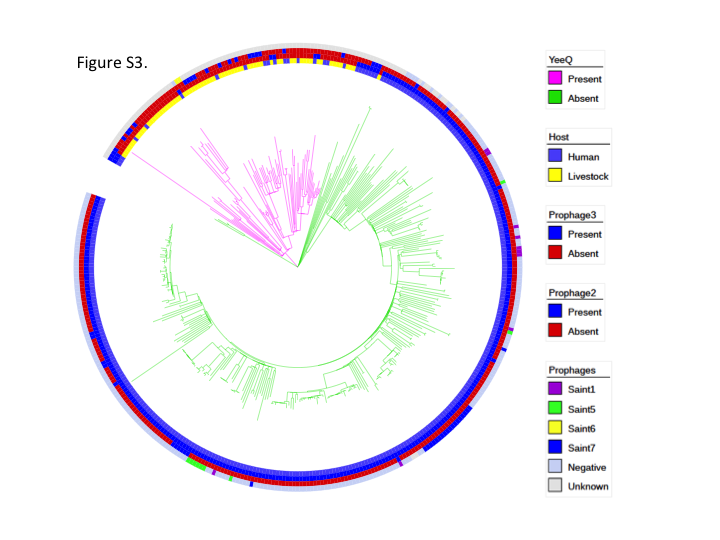

Supplement: FIG S3 [file mbo001173136sf3.tif]

## Frequency distribution

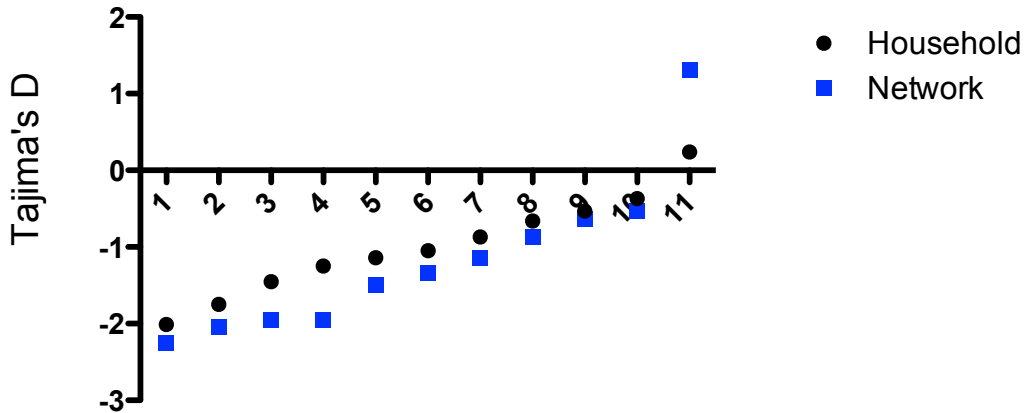

Supplement: FIG S4 [file mbo001173136sf4.pdf]
